# Impact of Oral Fast Release Amantadine on Movement Performance in Patients with Parkinson’s Disease

**DOI:** 10.3390/pharmaceutics2030313

**Published:** 2010-09-20

**Authors:** Siegfried Muhlack, Patricia Müsch, Sandra Konietzka, Dirk Woitalla, Horst Przuntek, Thomas Müller

**Affiliations:** Department of Neurology, St. Josef Hospital, Ruhr University Bochum, Gudrunstrasse 56, 44791 Bochum, Germany

**Keywords:** Parkinson’s disease, amantadine, motion, cognition

## Abstract

Application of oral fast release amantadine and levodopa may induce an improvement of motor symptoms in patients with Parkinson’s disease (PD). The objective of this trial was to investigate the clinical efficacy of a fast release amantadine sulfate formulation on simple and complex movement performance and putative relations to the pharmacokinetic behavior in PD patients. We challenged two cohorts of 12 PD patients, who were taken off their regular antiparkinsonian treatment for at least 12 hours, with oral 300 mg amantadine sulfate. We scored motor symptoms and performed instrumental tasks, which ask for performance of simple or complex motion series under cued conditions. Motor symptoms and performance of complex movements significantly improved in contrast to the carrying-out of simple motions. N-methyl-D-aspartic acid antagonistic and dopaminomimetic amantadine also influences altered higher predominant prefrontal cognitive functions. Therefore, performance of complex motion series improved, whereas carrying-out of simple repetitive movements is more associated to the striatal dopamine dependent basal ganglia function.

## 1. Introduction

The resurgence of amantadine as a treatment option for Parkinson’s disease (PD) occurred due to its therapeutic efficacy on dyskinesias and motor fluctuations, its association with increased survival in PD and putative neuroprotective effects due to its N-methyl-D-aspartic acid antagonistic properties [1,2]. Moreover, the known positive effects of amantadine on cognitive behavior may also influence performance of simple and complex movements. Simple standardized instrumental tests may measure these motions that are altered in PD patients [3]. One of these motor tests, peg insertion, asks for a performance of a complex movement series. This procedure demands for complex information processing with input of visuospatial cognition, self-elaboration of internal strategies, and sorting and planning—all of which are influenced by the modulatory role of striatal dopamine levels on association areas of the prefrontal cortex [3]. Our peg insertion procedure resembles another test, which demands performance of complex movements with an alternate tapping of the index and the middle fingers on two keys that are 20 cm apart [4]. The results obtained with this instrumental tool showed a close relationship to motor impairment and may be useful for serial longitudinal studies on progression of PD [4]. In contrast, a simultaneously evaluated tapping task, which simply asks for tapping with one finger on one key, did not show the same value. This motor test asks for repetitive performance and programming of standardized movements and requires low cognitive efforts, since the subject may create a fixed habit tendency with a consistent saving of cognitive resources. The individuals learn a standardized performance of a certain sequence of movements, which is based on an automatic function of a cognitive set [3]. We use a similar instrumental motor task for assessment of simple movements and motor impairment in PD, but this test was not sensitive enough to reflect improved motor symptoms after acute levodopa intake or daily 200 mg amantadine sulfate infusion during a three day interval. In contrast, the simultaneously performed more complex peg insertion test was sensitive enough [3].

The objective of this trial was to investigate the clinical efficacy of a novel fast release amantadine sulfate formulation on simple and complex movement performance and putative relations to the pharmacokinetic behavior in PD patients under standardized conditions. 

## 2. Experimental Section

### 2.1. Study design

The hospitalized PD patients only received 300 mg fast release amantadine sulfate at 7.00 a.m. after an overnight fasting without additional intake of their regular antiparkinsonian concomitant medication. We scored all PD patients at baseline and 30, 60, 90 and 120 minutes after oral amantadine intake with the part III (motor examination) of the Unified Parkinson’s Disease Rating Scale (UPDRS) from 7.00 to 9.00 a.m and then performed both instrumental tests. All participants were on identical standardized conditions until 9.00 a.m., in order to eliminate possible disturbing circumstances. 

### 2.2. Subjects

We enrolled 12 PD patients into the study (Table 1). Six of them showed higher UPDRS motor scores on the right side and five on the left side, while one patient showed the same score on both sides. CT- or MRI-scans showed no evidence of parenchymal lesion or atrophy in all participants. No individual was previously exposed to neuroleptic drugs. Exclusion criteria were clinical signs of dementia, electrophysiological or neuroradiologic evidence of additional CNS pathology exceeding PD. All patients fulfilled clinical diagnostic UK Brain bank criteria for PD. 

**Table 1:** The characteristics of the Parkinson’s disease patients.

N	sex	Age	height	weight	duration	MMSE	UPDRS	I	II	III	IV	HYS	DA	LD
1	1	62	178	85	7	25	45	2	14	28	1	3	-	0
2	2	63	168	73	2	29	35	3	8	24	0	1.5	40 mg DHEC	200
3	2	55	168	68.5	2	29	49	2	12	34	1	3	2.5 mg pergolide	300
6	2	65	162	71	10	30	21	0	5	15	1	1	-	500
5	1	66	1.69	80	2	30	34	1	8	24	1	1.5	-	0
7	1	46	178	76	7	30	23	0	9	13	1	1.5	3.75 mg pergolide	0
8	1	63	175	80	2	30	27	1	9	17	0	1.5	30 bromocriptine	400
4	1	79	172	87	5	25	65	3	24	37	1	2	0.36 pramipexole	400
5	2	66	159	53	15	30	45	1	13	30	1	1.5	3 mg pergolide	350
6	2	71	163	71	4	30	53	2	22	28	1	2	9 mg ropinirole	500
7	1	63	179	113	4	25	28	1	5	22	0	1.5	5 mg ropinirole	500
3	1	66	168	70	0.5	30	35	2	8	25	0	1.5	-	0

N = code of subject; sex : 1 male, 2 = female; age is given in years (age: 63.75 ± 7.97; 46–79 [mean ± SD; minimum–maximum]) years; duration = duration of PD in years (5.04 ± 4.17; 0.5–15); MMSE = Mini Mental State Examination Score (28.58 ± 1.19; 28–30); UPDRS = UPDRS total score (38.33 ± 13.28; 21–65); I = mental examination (1.5 ± 1; 0–3); II = UPDRS II (daily living activities) (11.42 ± 6.1); III = UPDRS III (motor examination) (24.75 ± 7.3; 13–37); IV = UPDRS IV (complication of therapy) (0.67 ± 0.49; 0–1), HYS = Hoehn and Yahr Scale (1.71 ± 0.5; I–III); UPDRS = Unified Parkinson’s Disease Rating Scale.

### 2.3. Instrumental tasks

We used two standardized instrumental procedures, peg insertion and tapping, in this study. 

### 2.4. Assessment of complex movements: peg insertion

We asked subjects to transfer 25 pegs (diameter 2.5 mm, length 5 cm) from a rack into one of 25 holes (diameter 2.8 mm) in a computer-based contact board individually and as quickly as possible. The distance between the rack and appropriate holes was 32 cm. The board was positioned in the center and the task was carried out on each side. When transferring each peg from rack to hole, elbows were allowed to be in contact with the table. We measured the time interval between insertion of the first and the last pin initially with the right- and then the left hand. We assessed the time period for this task by a computer to 100 ms accuracy. The peg insertion results represent the time of the task performance with the right and left hand in seconds. 

### 2.5. Assessment of simple movement: tapping

Individuals tapped as quickly as possible on a contact board (3 cm × 3 cm) with a contact pencil for a period of 32 seconds after the initial flash of a yellow stimulus light. We did not control for peak height reached by the pencil. The board was positioned in the center when the task was carried out on each side. When performing the task, elbows were allowed to be in contact with the table. We obtained the number of contacts by a computer. First we measured the frequency of tapping with the right and then with the left hand. The tapping rate represented the computed sum of tapping results of both hands. We allowed all participants to get familiar with both tasks for a time interval of 60 seconds to reduce or avoid learning and training effects on test performance on the day before the amantadine administration.

### 2.6. Blood samples

10 ml venous blood samples for estimation of amantadine plasma levels were taken from an antecubital vein through an indwelling cannula kept patient by an infusion of heparin in saline solution (10 U/mL). We performed venous puncture 20 minutes before the baseline investigation to enable stable conditions in particular for the following consecutive performance of the instrumental tests. Then blood samples were taken with motor assessment synchronously. Blood (3 mL) was drawn with a separate syringe and discarded before each 10 mL blood, which were placed in EDTA-test tubes. The plasma obtained from rapid centrifugation was immediately frozen at -80 ºC until analysis. We used reversed-phase high performance liquid chromatography in combination with electrochemical detection for the estimation of amantadine in plasma.

### 2.7. Statistics

Data showed a normal distribution according to the Kolmogorow-Smirnow test. As a result, we only performed parametric tests. We used ANCOVA with a repeated measures design and set age, sex, computed body mass index and the Hoehn and Yahr Stage as covariates. We employed Tukeys HSD test for the post hoc analysis. For the correlation analysis, we computed the various occurring changes with the formula: baseline value – value at 30 (60, 90, 120) minutes following amantadine intake and computed the corresponding Area under the curve values using the linear trapezoidal rule. 

## 3. Results and Discussion

### 3.1. Motor symptoms

UPDRS motor scores significantly (ANCOVA: F_(dF 4, dF 44) _= 27.22, p = 2.11E-11) reduced, accordingly the UPDRS III subscores for akinesia (ANCOVA: F_(dF 4, dF 44) _= 14.75, p = 1.02E-07), rigidity (ANCOVA: F_(dF 4, dF 44) _= 6.49, p = 0.0003) and tremor (ANCOVA: F_(dF 4, dF 44) _= 7.65, p = 9.03E-05) also significantly improved (Table 2). There was no impact of the covariates. 

**Table 2:** Comparisons of patients’ characteristics and pharmacokinetic results.

	Baseline	30 minutes	60 minutes	90 minutes	120 minutes
PIS	128.98 ± 16.64; 109.02 - 167.35	125.76 ± 17.85; 104.25 - 160.65	122.34 ± 17.62; 100.33 - 156.38	126.12 ± 16.84; 105.85 - 161.06	122.64 ± 15.63; 98.99 - 147.39
p		**ns**	**0.01**	**ns**	**0.01**
tapping	312.58 ± 41.84; 243 - 375	310.75 ± 45.57; 216 - 378	309.67 ± 53.33; 186 - 388	314.08 ± 50.36; 208 - 390	329.75 ± 38.13; 251 - 391
p		**ns**	**Ns**	**ns**	**ns**
UPDRS III	26.42 ± 7.95; 18 - 44	20.75 ± 8.29; 7- 33	20.25 ± 8.35; 7 - 33	20 ± 7.9; 7 - 32	19.92 ± 8.60; 6 - 33
p		**0.0001**	**0.0001**	**0.0001**	**0.0001**
akinesia	11.58 ± 2.15; 9 - 16	8.58 ± 3.18; 1 - 12	8.50 ± 3.03; 1 - 11	8.5 ± 3.06; 1 - 11	8.58 ± 2.81;1 - 11
p		**0.0001**	**0.0001**	**0.0001**	**0.0001**
rigidity	4.67 ± 3.58; 0 - 12	3.42 ± 3.42; 0 - 12	3.33 ± 3.23; 0 - 11	3 ± 3.3; 0 - 12	3.17 ± 3.33; 0 -12
		**0.012**	**0.007**	**0.0005**	**0.0018**
tremor	4.75 ± 3.08; 1 - 11	3.75 ± 2.45; 1 - 8	3.42 ± 2.61, 0 - 8	3.58 ± 2.54; 0 - 8	3.42 ± 2.64; 0 - 8
p		**0.009**	**0.0004**	**0.002**	**0.0004**

All data are shown as mean ± standard deviation, minimum – maximum; p- values represent post hoc comparisons against baseline; peg insertion results are given in seconds; tapping represents the rates within a period of 32 seconds; baseline, 30 minutes, 60 minutes, 90 minutes, 120 = timepoint 0, 30, 60, 90, 120 minutes after baseline; significant results are bold; UPDRS = Unified Parkinson’s Disease Rating Scale; III = motor examination (items 18 – 31), respectively computed subscores for akinesia, rigidity, tremor.

### 3.2. Instrumental motor tests

Peg insertion results significantly decreased (ANCOVA F_(dF 4, dF 44) _= 4.16, p = 0.006) after amantadine intake (Table 2), whereas tapping rates did not significantly change (ANCOVA F_(dF 4, dF 44) _= 1.95, p = 0.12). No impact of the covariates occurred.

### 3.3. Pharmacokinetics of amantadine

**Figure 1:**
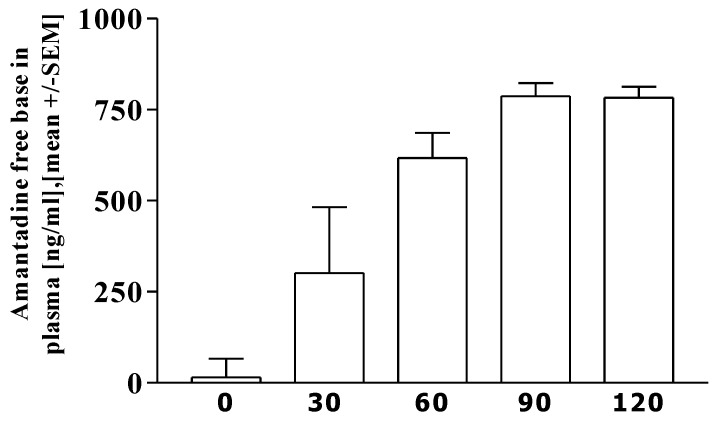
Pharmacokinetic data of free amantadine. Results are shown at moment 0, and 30, 60, 90, 120 minutes after receiving amantadine.

Figure 1 illustrates the increase of Amantadine free base in plasma. The AUC value _0–120 min_ was [mean] 63120 ± 16650.2 [SD], [range] 44550–88550 ng × min/mL. The maximum concentration C_max_ was 800.42 ± 143.13, 622.2–1060.1 ng/mL, the time to C_max_ (T_max_) was 85 ± 11.68, 60–90 minutes.

### 3.4. Correlation analysis

We found no significant associations between the pharmacokinetic and the clinical data (results not shown). 

### 3.5. Discussion

Our study confirms that administration of a fast release amantadine sulfate formulation alleviates bradykinesia, rigidity and tremor in treated PD patients in an open label fashion [3]. 

Our pharmacokinetic results show, that this fast release amantadine sulfate formulation is well absorbed after oral intake. In an earlier study, we found a relationship between pharmacokinetic data of levodopa and simple but not complex movement behavior [7]. The missing associations of the correlation analysis between the scored clinical improvement, the motor test outcomes and the pharmacokinetic results suggest that the antiparkinsonian efficacy of amantadine is indirectly triggered via other neurotransmitter systems, *i.e.* the glutamatergic one, and drug induced modification of receptors expression and is not directly influenced by striatal dopamine metabolism.

Only complex but not simple movement performance improved after acute amantadine administration, which confirms our findings with intravenous amantadine and levodopa [7]. Since the tapping procedure asks for repetitive carrying out and programming of standardized motions, it requires low cognitive efforts and thus low attention. This more autonomic functioning of the control of attention and processing of this selective attention is relatively intact in PD and may be associated to the precentral and postcentral gyri and supplementary motor area according to the outcomes of a PET study with repeat measurements of regional cerebral blood flow in younger subjects [8]. Therefore, we assume that our tapping outcomes did not improve after amantadine but after levodopa application and that the tapping procedure is associated with predominant striatal dopamine dependent function [7]. In contrast, the complex peg insertion procedure demands for a more complex sequence of aimed movements and thus additionally demands visuospatial cognition and further higher brain functions. However these efforts are influenced by the modulatory role of striatal dopamine levels on association areas of the prefrontal cortex. It is known, that cognitive weakness in PD patients may result from a dysfunction of dopaminergic pathways of mesial and dorsolateral prefrontal regions due to the dopaminergic deficit in the caudate according to [^18^F] Dopa positron emission tomography neuroimaging studies [9]. From this point of view, performance of complex movements is more sensitive to dopamine dependent prefrontal cognitive processes. Thus, this test hypothetically better reflects improvement of motor impairment in PD patients after application of dopaminergic and/or indirect dopaminomimetic drugs, like amantadine [3]. 

Amantadine also improves wakefulness, vigilance and cognitive processing, all of which may also hypothetically contribute to improved performance of peg insertion with its need for more cognitive load [10]. Thus, our study indirectly confirms a previous trial, which demonstrated a significant better performance of a complex choice reaction time paradigm but not a simple reaction time task after additional amantadine application in PD subjects of HYS I-III [10]. There was a missing significant improvement of peg insertion outcomes 90 minutes after amantadine intake according to the post hoc analysis. In view of the computed mean T_max_ of the amantadine plasma levels, we assume that the adaptation of the brain to the C_max_ of amantadine caused a temporary deterioration of cognitive function and movement abilities. This resulted in the temporarily increased peg insertion interval and the corresponding missing significant p value in the post hoc analysis.

A drawback of this trial is the missing comparison to the efficacy of placebo, which may also release striatal endogenous dopamine and therefore may hypothetically influence motor test outcomes and clinical rating scores [5,6]. Further shortcomings include the low number of participants and the relative short washout period of 12 hours, which is the usual interval similar to other trials of treated PD patients despite the half–life of dopamine agonists, like pergolide, pramipexole, *etc*. Generally, the whole protocol was rather demanding for the participants and a longer washout period is not ethical and not tolerated by PD patients. 

## 4. Conclusion

Amantadine also improves performance of complex motion series due to a putative positive impact on secondary associated brain areas, whereas carrying out of simple movements with their demand for low cognitive efforts is more associated to striatal dopamine dependent basal ganglia function [3,10]. 

## References

[1] Metman L.V., Del Dotto P., LePoole K., Konitsiotis S., Fang J., Chase T.N. (1999). Amantadine for levodopa-induced dyskinesias: a 1-year follow-up study. Arch. Neurol..

[2] Thomas A., Iacono D., Luciano A.L., Armellino K., Di Iorio A., Onofrj M. (2004). Duration of amantadine benefit on dyskinesia of severe Parkinson's disease. J. Neurol. Neurosurg. Psychiat..

[3] Müller T., Kuhn W., Schulte T., Przuntek H. (2003). Intravenous amantadine sulphate application improves the performance of complex but not simple motor tasks in patients with Parkinson's disease. Neurosci. Lett..

[4] Pal P.K., Lee C.S., Samii A., Schulzer M., Stoessl A.J., Mak E.K., Wudel J., Dobko T., Tsui J.K. (2001). Alternating two finger tapping with contralateral activation is an objective measure of clinical severity in Parkinson's disease and correlates with PET. Parkinsonism Relat. Disord..

[5] Fuente-Fernandez R., Stoessl A.J. (2004). The biochemical bases of the placebo effect. Sci. Eng. Ethics.

[6] Goetz C.G., Laska E., Hicking C., Damier P., Müller T., Nutt J., Warren O.C., Rascol O., Russ H. (2008). Placebo influences on dyskinesia in Parkinson's disease. Movement Disord..

[7] Muhlack S., Konietzka S., Woitalla D., Przuntek H., Muller T. (2004). Simple movement sequences better correlate to levodopa plasma levels than complex ones. J. Neural Transm. Suppl..

[8] Carey L.M., Abbott D.F., Egan G.F., Tochon-Danguy H.J., Donnan G.A. (2000). The functional neuroanatomy and long-term reproducibility of brain activation associated with a simple finger tapping task in older healthy volunteers: a serial PET study. Neuroimage.

[9] Andreasen N.C., Cohen G., Harris G., Cizadlo T., Parkkinen J., Rezai K., Swayze V.W. (1992). Image processing for the study of brain structure and function: problems and programs. J. Neuropsychiatr. Clin. Neurosci..

[10] Pinter M.M., Birk M., Helscher R.J., Binder H. (1999). Short-term effect of amantadine sulphate on motor performance and reaction time in patients with idiopathic Parkinson's disease. J. Neural Transm..

